# Investigation of the interactions between Pt(II) and Pd(II) derivatives of 5,10,15,20-tetrakis (*N*-methyl-4-pyridyl) porphyrin and G-quadruplex DNA

**DOI:** 10.1007/s00775-015-1325-8

**Published:** 2016-01-09

**Authors:** Navin C. Sabharwal, Oscar Mendoza, John M. Nicoludis, Thomas Ruan, Jean-Louis Mergny, Liliya A. Yatsunyk

**Affiliations:** Department of Chemistry and Biochemistry, Swarthmore College, 500 College Ave., Swarthmore, PA 19081 USA; INSERM U1212, CNRS, 33600 Pessac, France; Université de Bordeaux, Bordeaux, France; Department of Chemistry and Chemical Biology, Harvard University, 12 Oxford St., Cambridge, MA 02138 USA; Lerner College of Medicine, Cleveland Clinic, 9500 Euclid Ave., Cleveland, OH 44195 USA

**Keywords:** G-quadruplex DNA, Metalloporphyrin, Light switch effect, Fluorescence, Human telomeric DNA

## Abstract

**Abstract:**

G-quadruplexes are non-canonical DNA structures formed by guanine-rich DNA sequences that are implicated in cancer and aging. Understanding how small molecule ligands interact with quadruplexes is essential both to the development of novel anticancer therapeutics and to the design of new quadruplex-selective probes needed for elucidation of quadruplex biological functions. In this work, UV–visible, fluorescence, and circular dichroism spectroscopies, fluorescence resonance energy transfer (FRET) melting assays, and resonance light scattering were used to investigate how the Pt(II) and Pd(II) derivatives of the well-studied 5,10,15,20-tetrakis(*N*-methyl-4-pyridyl)porphyrin (TMPyP4) interact with quadruplexes formed by the human telomeric DNA, Tel22, and by the G-rich sequences from oncogene promoters. Our results suggest that Pt- and PdTMPyP4 interact with Tel22 via efficient *π*–*π* stacking with a binding affinity of 10^6^–10^7^ M^−1^. Under porphyrin excess, PtTMPyP4 aggregates using Tel22 as a template; the aggregates reach maximum size at [PtTMPyP4]/[Tel22] ~8 and dissolve at [PtTMPyP4]/[Tel22] ≤ 2. FRET assays reveal that both porphyrins are excellent stabilizers of human telomeric DNA, with stabilization temperature of 30.7 ± 0.6 °C for PtTMPyP4 and 30.9 ± 0.4 °C for PdTMPyP4 at [PtTMPyP4]/[Tel22] = 2 in K^+^ buffer, values significantly higher as compared to those for TMPyP4. The porphyrins display modest selectivity for quadruplex vs. duplex DNA, with selectivity ratios of 150 and 330 for Pt- and PdTMPyP4, respectively. This selectivity was confirmed by observed ‘light switch’ effect: fluorescence of PtTMPyP4 increases significantly in the presence of a variety of DNA secondary structures, yet the strongest effect is produced by quadruplex DNA.

**Graphical abstract:**

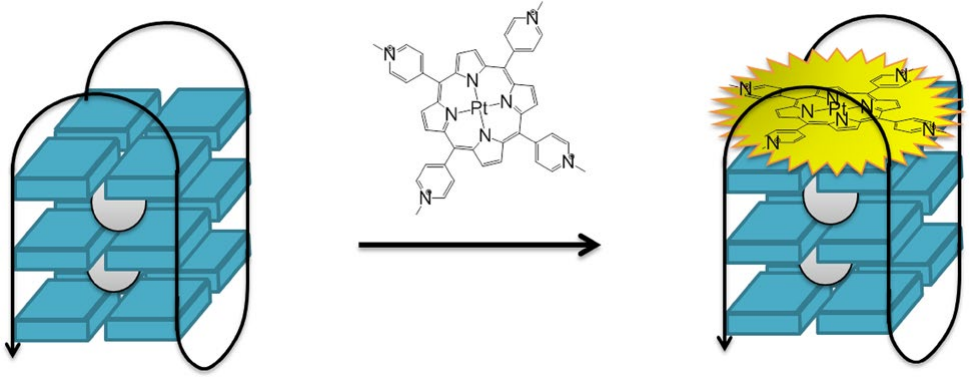

**Electronic supplementary material:**

The online version of this article (doi:10.1007/s00775-015-1325-8) contains supplementary material, which is available to authorized users.

## Introduction

**Fig. 1:**
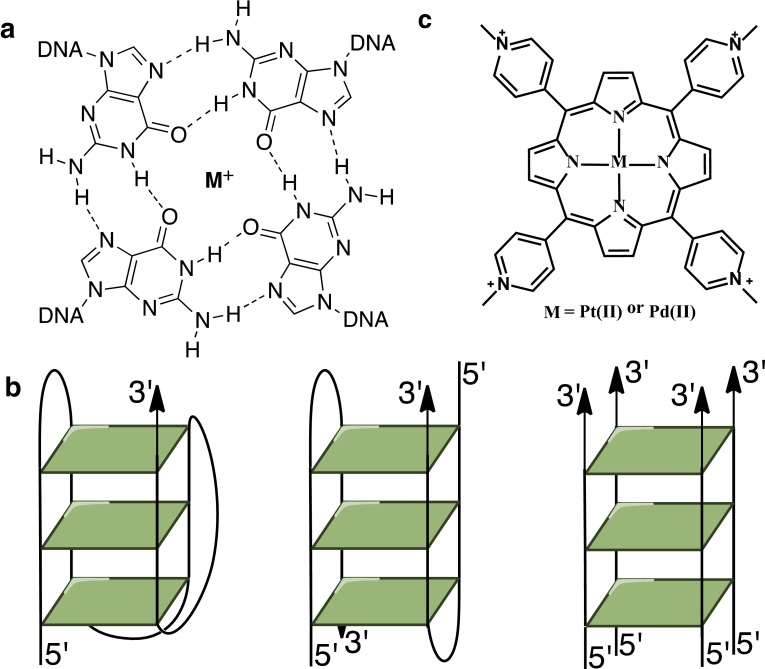
**a** G-tetrad consists of four guanine residues associated together through Hoogsteen hydrogen bonding and stabilized by a monovalent cation. **b** Schematic representation of a monomolecular mixed-hybrid GQ (*left*), bimolecular antiparallel GQ (*middle*) and tetrastranded parallel GQ (*right*). **c** Structures of PtTMPyP4 and PdTMPyP4

In addition to a Watson–Crick duplex, DNA can exist in a variety of non-canonical secondary structures, including G-quadruplex DNA [1]. G-quadruplexes (GQs) are formed by *π*–*π* stacking between G-quartets composed of four guanines connected via Hoogsteen hydrogen bonding and stabilized by a coordinating cation (Fig. 1a, b). Sequences with quadruplex-forming potential are found throughout the human genome, notably at telomeres [2] and at the promoters of many oncogenes [3–5]. GQs may play an important role in a variety of biological processes such as telomere maintenance, transcription, translation, replication, genome stability, and DNA repair [6–9]. More importantly, the existence of GQs in living cells was recently validated by their direct visualization [10–12]. G-rich DNA is now established as a potent therapeutic target, especially for cancer.

Telomeres are present at the end of eukaryotic chromosomes and protect them from degradation and end-fusion. They comprise repetitive G-rich sequences, TTAGGG in humans and other vertebrates. Realization that human telomeric DNA has a potential to fold into a quadruplex structure brought it to the forefront of drug discovery efforts. Formation of quadruplexes in telomeres leads to inhibition of telomerase, the enzyme responsible for telomere maintenance and, hence, immortality of cancer cells. The most widely studied model of human telomeric DNA is Tel22, AGGG(TTAGGG)_3_, whose structure, folding, and stability have been researched extensively [13]. This oligonucleotide or its variants form a unimolecular antiparallel GQ in Na^+^ buffer [14], at least two different mixed-hybrid structures in K^+^ buffers [15–17], and a parallel GQ when crystallized in the presence of K^+^ [18] or in solution under dehydrating conditions (e.g., PEG or ethanol) [19].

Structure and stability of quadruplexes and, hence, their biological functions can be altered by the presence of small molecule ligands [20–23]. Ligands induce quadruplex formation or stabilize existing quadruplexes in telomeres and in oncogene promoters, leading to the inhibition of telomerase and to an alteration of oncogene expressions. Selective interactions between ligands and quadruplex DNA constitute a novel anticancer methodology. Over the last 18 years, a number of drug-discovery research programs have produced successful GQ-selective ligands [22, 24–27], many of which have been shown to inhibit tumors in vitro and in vivo. Our work here focuses on two metal derivatives of the widely studied ligand 5,10,15,20-tetrakis(*N*-methyl-4-pyridyl) porphyrin, TMPyP4, with Pt(II) and Pd(II) (Fig. 1c). TMPyP4 was shown to inhibit telomerase [28–30] and downregulate expression of oncogenes *c*-*myc* [3] and *Kras* [31] through binding, stabilization, and structural alteration of quadruplexes. Cellular uptake and localization studies demonstrate that TMPyP4 and its derivatives can accumulate rapidly in nuclei of normal and tumor cells [32] at levels sufficient for tumor growth arrest yet non-toxic to somatic cells [30] without appreciable membrane binding. In addition, TMPyP4 is water soluble and brightly colored, which facilitates its administration and study. Although TMPyP4 is an excellent quadruplex stabilizer, it suffers from modest selectivity for GQ vs. duplex DNA [33]. In spite of this limitation, TMPyP4 is still widely used by researchers in the quadruplex field, with over 110 publications in the last five years (according to http://www.gopubmed.com).

In order to utilize TMPyP4′s excellent quadruplex stabilizing ability, improve its GQ-selectivity, and confer additional useful properties such as fluorescence, a variety of modifications to TMPyP4 were reported [24, 25]; here we focus on metalation. In principle, a variety of metals can be placed in the center of TMPyP4; many of these metal derivatives were shown to bind quadruplexes, notably those with Cu(II), Zn(II), Ni(II), Mn(III), In(III), and Co(III) [25, 34–40]. Metalation of TMPyP4 changes its electronic structure, leading to drastic changes in its ability to bind GQ DNA and inhibit telomerase [25]. The presence of a metal decreases the electron density of the ligand’s aromatic system, increasing its *π*–*π* stacking ability with the terminal G-tetrad, leading to stronger ligand–GQ interactions. In addition, if a metal coordinated to a ligand is placed above the center of the G-tetrad, it can replace the monovalent ion found there, strengthening ligand–GQ interactions, due to electrostatic effect. This mode of binding was observed between the human telomeric DNA and Ni(II) or Cu(II) Salfen ligands [41]. Finally, central metal substitution into TMPyP4 may introduce axial ligands to the metal, as is the case for Zn(II) (one axial water) or Mn(III) and Co(III) (two axial water molecules), further affecting its quadruplex binding abilities and importantly, the selectivity.

The goal of this report was to investigate how PtTMPyP4 and PdTMPyP4 interact with quadruplex structures and how these interactions differ from those with the parent porphyrin, TMPyP4. This comparison will allow us to highlight the specific roles that metal ions may play in the interactions between ligands and quadruplexes. Both porphyrins adopt square planar geometry similar to that of TMPyP4; they are water soluble, intensely colored and fluorescent, providing convenient handles on monitoring their interactions with DNA. PtTMPyP4 was reported to inhibit telomerase (although less efficiently as compared to TMPyP4) [25, 29] and act as photosensitizer in photodynamic therapy [42]. We performed UV–Vis, CD, and fluorescence titrations, FRET melting assay and resonance light scattering studies using quadruplex structures formed by human telomeric DNA and DNA from a variety of biologically relevant oncogene promoters. In many respects, both Pt(II) and Pd(II) metallated derivatives of TMPyP4 resemble their parent porphyrin molecule but display significantly stronger quadruplex stabilization and higher fluorescence. Our findings have significant implication for understanding the exact role that metals play in ligand binding to quadruplex DNA.

## Materials and methods

### Porphyrins, oligonucelotides, and buffers

PtTMPyP4 was a generous gift from Dr. Robert F. Pasternack, Swarthmore College, and PdTMPyP4 was purchased from Frontier Scientific (Logan, UT, USA). Porphyrin stock solutions were prepared in double-distilled water (ddH_2_O) at 1.0–1.4 mM concentrations and stored at −20 °C in the dark. Porphyrin concentrations were determined via UV–Vis spectroscopy, using *ε*_401_ = 1.72 × 10^5^ M^−1^cm^−1^ for PtTMPyP4 [43] and *ε*_418_ = 1.68 × 10^5^ M^−1^cm^−1^ for PdTMPyP4 [44].

Oligonucleotides were purchased from Midland Certified Reagent Company (Midland, TX, USA) or Eurogentec (Liège, Belgium); calf thymus (CT) DNA was obtained from Sigma-Aldrich; the fluorescently labeled oligonucleotide 5′-6-FAM-GGG(TTAGGG)_3_-Dabcyl-3′ (F21D) was purchased from IDT (Coralville, IA, USA). All oligonucleotides were dissolved at 1.0 mM strand concentration, while F21D was dissolved at 0.1 mM in ddH_2_O and stored at −80 °C. To induce the formation of the most thermodynamically stable GQs, oligonucleotides were heated at 90 °C for 5 min, cooled to room temperature over the course of 3–4 h, and incubated overnight at 4 °C. CT DNA was solubilized in ddH_2_O at about 1 mM with gentle mixing during 1 week at 4 °C. The solution was then filtered, stored at 4 °C, and used within 6 months. DNA concentration was determined via UV–Vis spectroscopy using extinction coefficients provided by the manufacturers and listed in Table 1. The following buffers were used in this study: 10 mM lithium cacodylate, pH 7.2, 5 mM KCl, 95 mM LiCl (5K); 10 mM lithium cacodylate, pH 7.2, 50 mM NaCl, 50 mM LiCl (50Na); 10 mM KPi, pH 7.0, 50 mM KCl (KPi).

**Table 1:** Oligonucleotide sequences, extinction coefficients and fluorescence enhancement data

Name	Sequence 5′ → 3′	ɛ_260,_ mM^−1^ cm^−1^	Fluorescence enhancement^a,b^
F21D	5′-6-FAM-GGG(TTAGGG)_3_-Dabcyl-3′	247.6	N/A
CT	Genomic calf thymus DNA	12.2 (per bp)	22.3 ± 0.6
17A	CCAGTTCGTAGTAACCC	160.9	38.1 ± 1.5
17B	GGGTTACTACGAACTGG	167.4	43.9 ± 3.0
17AB	Duplex formed by 17A and 17B oligonucleotides	328.3	46.0 ± 0.7
ds26	CAATCGGATCGAATTCGATCCGATTG	253.2	39.1 ± 5.2
Tel22	AGGGTTAGGGTTAGGGTTAGGG	228.5	54.2 ± 14.6 (50Na buffer) 71.8 ± 10
VEGF	GGGAGGGTTGGGGTGGG	171.4	76.9 ± 3.8
cMyc	TGAGGGTGGGTAGGGTGGGTAA	228.7	28.0 ± 5.0
26TelG4	AGGGGTTAGGGGTTAGGGGTTAGGGG	268.9	54.1 ± 15 (50Na buffer) 65.4 ± 4.8
Bcl-2	GGGCGCGGGAGGGAATTGGGCGGG	237.4	39.2 ± 5.7
cKit1	GGGAGGGCGCTGGGAGGAGGG	213.2	28.0 ± 5.6
G4TERT	AGGGGAGGGGCTGGGAGGGC	202.9	85.7 ± 0.9
TBA	GGTTGGTGTGGTTGG	143.3	32.6 ± 6.8 (50Na buffer) 29.5 ± 5.4
G_4_T_4_G_4_	GGGGTTTTGGGG	115.2	40.5 ± 12 (50Na buffer) 42.2 ± 9.2

^a^Fluorescence enhancement is defined as a ratio of the fluorescence of PtTMPyP4 in the complex with the specified DNA to the fluorescence of the porphyrin alone

^b^5K buffer was used for fluorescence enhancement studies unless stated otherwise

### UV–Vis studies

A Cary 300 Varian spectrophotometer with a Peltier-thermostated cuvette holder (error of ±0.3 °C) or UVIKON XL or XS spectrophotometers were used for all UV–Vis studies. Samples were prepared in 1 cm quartz cuvettes or in 1 cm methacrylate cuvettes; the latter were used to minimize porphyrin adsorption to the surface of a cuvette.

#### Aggregation studies

UV–Vis spectra were collected as a function of porphyrin concentration in the spectral window of 350–450 nm in water for PtTMPyP4 (0.5–20 µM range) and in 5K buffer for PdTMPyP4 (0.5–50 µM range).

#### Titrations of PtTMPyP4 and PdTMPyP4 with Tel22 in 5K buffer

Titrations were performed by stepwise additions of Tel22 to a solution of 1–6 µM PtTMPyP4 or ~3 µM PdTMPyP4 in 5K buffer. Solution of Tel22 was added to a cuvette with porphyrin, mixed thoroughly and equilibrated for two min after which UV–Vis spectra were acquired in 350–650 nm range. Titration was deemed complete when the spectra collected after three successive additions of Tel22 were nearly superimposable. Data were treated as described in our earlier work [36]. Additionally, the spectra were corrected for dilution effect. Both direct fitting of UV–Vis data (assuming two-state equilibrium) and Scatchard analysis were used to obtain the values of binding affinity and stoichiometry. Hypochromicity (% H, decrease in signal intensity) and red shift (change in peak position) were extracted from UV–Vis data. PtTMPyP4 titrations were also carried out in KPi buffer, and the results are presented in Supplementary Material. Titrations were done in triplicate.

#### Continuous variation analysis: Job’s plot [45]

Solutions of PtTMPyP4 and of Tel22 were prepared in 5K buffer at equal concentrations of ~3 µM. Two sets of experiments were completed. In the first one, samples of PtTMPyP4 were placed in the sample and in the reference cells of UV–Vis spectrophotometer and were titrated with Tel22 (sample cell) and with 5K buffer (reference cell) in identical manner. In the second set of experiments, a solution of Tel22 was placed in the sample cell and a solution of 5K was placed in the reference cell; both cells were titrated with PtTMPyP4 solution. The difference spectra were collected in 350–670 nm range. Job plots were constructed by plotting the absorbance at selected wavelengths (where the largest change was observed) vs. mole fraction of the porphyrin (range 0–1). Minima or maxima on Job plots report on binding stoichiometry. Similar experiments were also completed in KPi buffer (see Supplementary Material).

### Fluorescence studies

#### Resonance light scattering (RLS)

RLS method allows us to study aggregation of chromophores using a conventional fluorimeter [46], a Spex Fluorolog-3 (HORIBA Jobin–Yvon, France) in our case. A solution of 2.5 mL of 5.1 µM PtTMPyP4 in KPi was titrated with 120 µM Tel22 (which also contained 5.1 µM PtTMPyP4 to avoid porphyrin dilutions) at 25 °C in a 1 cm cuvette. Time scans were collected first to determine the amount of time required for signal stabilization (~15 min). The following parameters were used for the time scans: a time increment of 2 s, an integration time of 1 s and slit widths of 1 nm for both excitation and emission. After signal stabilization (for 15 min), synchronous scans were collected with the following parameters: excitation at 460 nm, emission range of 300–600 nm, offset of 0 nm, increment of 0.5 nm, averaging time of 0.5 s, number of scans 2 (averaged), and 1 nm slits both for excitation and emission. Before each new addition of Tel22, the UV–Vis spectra were also collected.

#### Fluorescence titrations

Fluorescence titrations were performed on a Photon Technology International QuantaMaster 40 spectrofluorimeter. A 2.0 mL solution of 1.7–2.0 µM PtTMPyP4 in 5K buffer was titrated with 60–100 µM of either Tel22 or CT DNA in a 1 cm methacrylate cuvettes with four transparent windows. After addition of DNA, the solution was thoroughly mixed, equilibrated for 30 s and emission scans were collected with the following parameters: excitation at 412 nm, emission range of 600–800 nm, increment of 1 nm, integration time of 0.5 s, slit of 5 nm each, and temperature of 20 °C. DNA was added until no further increase of fluorescence was observed.

#### Light-switch effect of PtTMPyP4 in the presence of a variety of DNA secondary structures

These experiments were carried out in triplicate in 96-well plates (Greiner Bio-one; 96-well, black, flat bottom) at 25 °C using Tecan Infinite M1000 PRO microplate reader. Every replicate contained a 50 µL of 0.11 µM solution of PtTMPyP4 to which 5 µL of 55 µM DNA was added; final DNA to porphyrin ratio was 50:1 to ensure that all porphyrin was saturated with DNA. The plate was stirred for 30 s and briefly centrifuged. Fluorescence emission was then recorded with the excitation wavelength set at 412 nm and the emission wavelength at 670 nm. Fluorescence enhancement was calculated as a ratio of fluorescence of PtTMPyP4/DNA mixture to the fluorescence of PtTMPyP4 alone. The DNA sequences used in this experiment are listed in Table 1.

#### Pt–Pt charge transfer band by fluorescence

A 3D excitation/emission fluorescence scan was performed on a sample containing 5.0 µM Tel22 and 30 µM PtTMPyP4 in 5K buffer. The 3D surface was built using individual emission scans in the range from 700 to 850 nm with excitation wavelength dimension covering the range from 500 to 680 nm (2 nm bandwidth).

#### Fluorescent resonance energy transfer melting studies, FRET

FRET studies were performed according to a well established protocol [47] using fluorescently labeled human telomeric DNA (F21D) in 5K buffer. CT DNA, a duplex competitor, was used in competition experiments, and unlabeled Tel22 was used as a control to assure that observed fluorescent changes are not due to contribution from the porphyrin. The specific experimental details and data treatment methods are described in our earlier work [36].

### Circular dichroism

Circular dichroism (CD) experiments were performed on either a Jasco J-800 or AVIV 410 spectropolarimeters containing a Peltier heating unit (error of ±0.3 °C) with a 1 cm quartz cuvette. On Jasco J-800, scans were collected at 100 nm/min rate, 1 nm increment, 1 s response time. Each final spectrum was obtained by averaging three scans, subtracting the spectrum of a buffer, and smoothing the data using a Savitzky–Golay function. Additional details can be found elsewhere [36].

#### CD annealing experiments

Solutions of Tel22, c-myc, or G4TERT at 2.5 µM were annealed either alone or with 5.0 µM PtTMPyP4 in 5K buffer, allowed to cool to room temperature, and equilibrated at 4 °C overnight. CD scans were then collected as described above.

#### CD titration experiments

A 2 µM solution of annealed quadruplex DNA was titrated with an increasing amount of PtTMPyP4 at 4 °C. After each addition of porphyrin, solution was thoroughly mixed and incubated for 3 min after which CD spectrum was collected and treated as describe above. PtTMPyP4 was added up to 4 equivalents. All data in this work were processed with Origin 9.0 software.

## Results and discussion

In this work, we investigated how Pt(II) and Pd(II) metalation of the widely studied quadruplex ligand TMPyP4 affects its interaction with quadruplex DNA from telomeres and oncogene promoters. In both porphyrins, PtTMPyP4 and PdTMPyP4, metal centers are expected to be four coordinate square planar without axial ligands and, thus, suitable for interaction with a terminal G-tetrad of a quadruplex. Under our experimental conditions, both porphyrins exist in a monomeric form (Figure S1), which simplifies our biochemical studies.

### Porphyrin-Tel22 binding explored through UV–Vis titrations

**Fig. 2:**
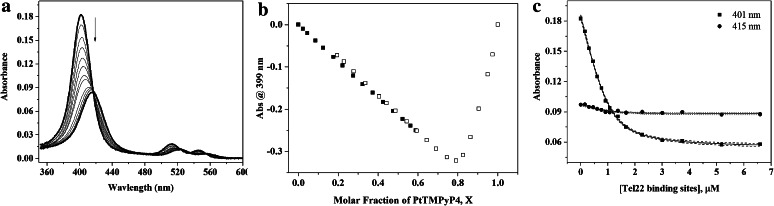
Titration of PtTMPyP4 with Tel22 in 5K buffer at 20 °C. **a** Representative UV–Vis absorption spectra of 1.1 μM PtTMPyP4 titrated with 22.0 μM Tel22. The final [GQ]/[PtTMPyP4] ratio was 0.93. **b** A representative Job plot constructed by plotting the difference in the absorbance values at 399 nm versus mole fraction of PtTMPyP4. The data from two titrations are shown starting with either DNA (*solid symbols*) or porphyrin (*open symbols*). **c** Direct fit of the titration data at specified wavelengths. Binding constant was determined to be (5.8 ± 0.8) × 10^6^ M^−1^ with a binding ratio of 7:1 porphyrin-to-GQ based on two independent experiments. *Dashed lines* represent 95 % confidence interval

UV–Vis titrations provide a convenient way to quantitatively characterize GQ–porphyrin interactions. Specifically, binding mode, stoichiometry, and binding constants can be determined via analysis of UV–Vis titration data. Representative UV–Vis titrations for Pt- and PdTMPyP4 are shown in Fig. 2 and in SI Figure S2, respectively. Hypochromicities were determined to be 53 ± 1 % and 45 ± 2 %, and red shifts were determined to be 13.5 ± 0.5 and 14 ± 1 nm for Pt- and PdTMPyP4, respectively. Similar values of red shift and hypochromicity were reported for Pt- and PdTMPyP4 binding to plasmid DNA (B-form pBluescript II plasmid) [48]. These high numbers might suggest intercalation as the possible binding mode based on extensive studies of porphyrin intercalation into duplex DNA [43]. However, this binding mode, although suggested by dynamic molecular modeling simulations [49], has not been observed experimentally. In structural studies, porphyrins bind to external G-tetrads (end-stacking) [20], loops, or to base-pairs [50], but do not intercalate. Regardless of the exact mode of binding, high values of hypochromicity and red shift indicate strong stacking interactions between the porphyrin and the DNA bases, which then perturb the electronic distribution around porphyrin ring. Loop or groove binding, most likely, would not produce such large changes in UV–Vis spectra, suggesting end-stacking as a binding mode.

To extract binding affinities and stoichiometries, UV–Vis data were subjected to a Direct Fit, which is based on the two-state equilibrium between free and quadruplex-bound porphyrin [36], and a Scatchard analysis, developed for a ligand bound to a long polyanionic DNA (notably duplex DNA). Because a quadruplex cannot be truly approximated as a long DNA, the results of Scatchard treatment need to be interpreted with caution and should be only used in conjunction with other data analyses. According to both models, if multiple binding sites exist on the DNA these binding sites are equivalent and non-cooperative. In other words, it is assumed that there is no preference of the porphyrin to one Tel22 binding site over another, and that the binding of one porphyrin does not affect the binding of a subsequent molecule. Thus, the binding ratios represent the total number of porphyrins bound and binding constants reflect all the binding events. Direct Fit of the data yielded a binding affinity of (5.8 ± 0.8) × 10^6^ M^−1^ for PtTMPyP4 when the binding stoichiometry was fixed at 7:1 porphyrin-to-DNA and (1.0 ± 0.3) × 10^7^ M^−1^ for PdTMPyP4 when the binding stoichiometry was fixed at 6.5 (Figs. 2c, S2c). Using Scatchard analysis of titration data for PtTMPyP4 we obtained a stoichiometry of 6.7 ± 0.5 and a binding affinity of (9.2 ± 0.1) × 10^6^ M^−1^; the same numbers for PdTMPyP4 are 6.5 ± 0.5 and (0.78 ± 0.03) × 10^7^ M^−1^. Both Direct Fits and Scatchard analyses are in excellent agreement with each other.

UV–Vis titration experiments were also conducted in KPi buffer, Figure S3, yielding 13.8 ± 0.3 nm red shift and 45 ± 2 % hypochromicity, values similar to those reported above for 5K buffer. Scatchard and Direct Fit analyses yielded ~7:1 porphyrin-to-DNA binding stoichiometry and (1–3) × 10^8^ M^−1^ binding affinity, higher as compared to the values in 5K buffer. This apparent discrepancy could be explained by the difference in ionic strength between KPi buffer (~60 mM) and 5K buffer (110 mM). Usually, increase in the ionic strength leads to weakening of interactions especially those with significant electrostatic component as is expected from quadruplex binding to PtTMPyP4 ligand that has 4+ positive charge.

Observed binding stoichiometry was also measured in the model independent Continuous Variation Analysis method, also known as Job plot method [45]. By keeping the total concentration of porphyrin and DNA constant and, at the same time, varying the mole fraction of the porphyrin and DNA, the binding ratio can be determined from the difference in absorbance as shown in Fig. 2b for 5K buffer and Figure S4 for KPi buffer. Job’s method is effective in identifying the binding ratio in a non-cooperative system as well as the overall binding ratio. With high enough resolution, other equilibria and their binding ratios can also be identified. Job plot analysis of the data using 399 nm wavelength yielded binding stoichiometry of 4:1 PtTMPyP4:Tel22. Interestingly and reproducibly, the data from the same Job plot titration at 425 nm (absorption maximum of a PtTMPyP4-Tel22 complex) yielded binding stoichiometry of only 2:1. In both cases, binding stoichiometry is lower than that obtained from the Direct Fit or Scatchard analysis of UV–Vis titrations. Our data can be reconciled by a model where 2–4 molecules of PtTMPyP4 bind Tel22 with high affinity, following by additional weaker binding of another 3–4 porphyrins. This second step could signify (1) binding of porphyrins to additional binding sites on Tel22, which is rather difficult to imagine; (2) non-specific binding between negatively charged DNA and positively charged porphyrin molecule; yet, reproducible binding stoichiometry argues against this case; or (3) stacking of additional porphyrins onto already bound porphyrin molecules (aggregation).

To test this latter possibility, we searched for MMLCT, metal–metal-to-ligand-charge transfer bands (usually in the near-infrared region of >700 nm) [51, 52] between Pt(II) ions in the stacked porphyrin complexes. While the MMLCT band was not observed, stacking of porphyrins without direct and close Pt–Pt interaction is still possible in a manner allowing for an efficient overlap of their aromatic systems. The presence of porphyrin aggregates was further investigated in resonance light scattering experiments.

### Resonance light scattering (RLS)

**Fig. 3:**
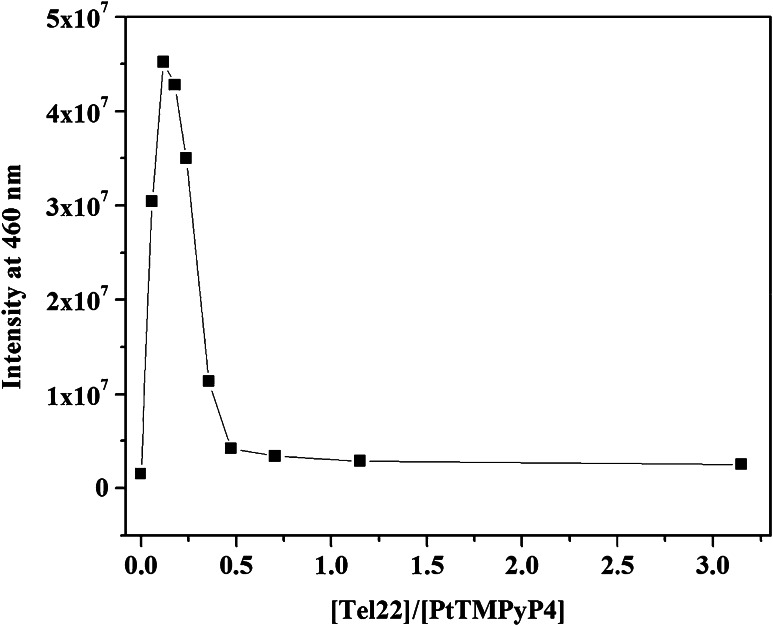
Resonance light scattering titration of PtTMPyP4 with Tel22. A solution of PtTMPyP4 at 5.1 µM in KPi buffer was titrated with 120 µM solution of Tel22 at 20 °C

RLS identifies the presence of supramolecular assemblies by detecting the amount of light scattered by them [46]. RLS takes advantage of the enhanced Rayleigh scattering of porphyrins near their absorption maximum (Soret band) in aggregated state. It is important to note that, unlike UV–Vis and fluorescence titrations (see below), RLS is performed under conditions of porphyrin excess. A ~5 µM solution of PtTMPyP4 was excited at 460 nm, and the scattering was measured at the same wavelength. In the absence of Tel22, PtTMPyP4 does not aggregate, which is in agreement with our UV–Vis aggregation studies (Figure S1). However, addition of Tel22 leads to aggregate formation, but only at low [Tel22]/[PtTMPyP4] ratios (Fig. 3). Specifically, aggregate size grows (as judged by the scattering intensity) and reaches maximum at [Tel22]/[PtTMPyP4] = 0.12, then aggregates decrease in size and disappear at [Tel22]/[PtTMPyP4] > 0.5. The RLS data suggest that the largest aggregates of PtTMPyP4-Tel22 contain ~8 molecules of PtTMPyP4 per each Tel22 molecule. When more DNA is available, these aggregates dissolve. The observed [PtTMPyP4]/[Tel22] ratio of 8 is in agreement with Scatchard analysis of UV–Vis titration data and the Direct Fit model suggesting 7:1 stoichiometry. Combining RLS and UV–vis data, we can propose the following binding mode for PtTMPyP4 and Tel22. PtTMPyP4 originally end-stacks onto Tel22 and, in the absence of sufficient Tel22 molecules, additional porphyrin molecules aggregate with each other stacking on the top of the already bound PtTMPyP4. Once more Tel22 becomes available, these aggregated dissolve and completely disappear at [Tel22]/[PtTMPyP4] > 0.5. Note, most of the experiments in this paper were performed at [Tel22]/[PtTMPyP4] > 0.5 where no aggregation was observed.

### Porphyrin-Tel22 binding and selectivity explored through fluorescence titrations

**Fig. 4:**
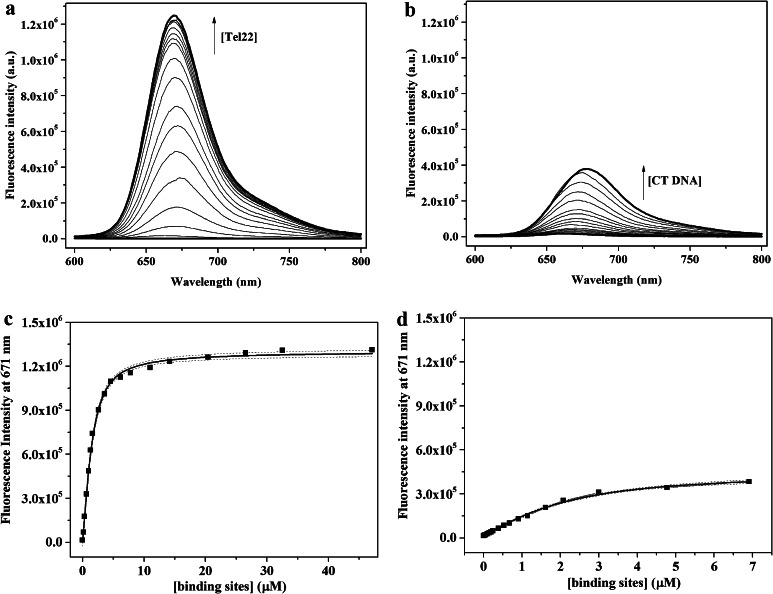
Fluorescent titration of PtTMPyP4 with DNA in 5K buffer at 20 °C. **a**, **b** Representative fluorescence titrations spectra for PtTMPyP4 upon addition of Tel22 and CT DNA. **c**, **d** Increase in fluorescence signal intensity at 671 nm as a function of DNA concentration and corresponding data fit for Tel22 and for CT DNA. Concentration of binding sites corresponds to concentration of DNA multiplied by the binding stoichiometry. Dashed lines represent 95 % confidence interval

PtTMPyP4 fluoresces weakly alone. Addition of a quadruplex DNA leads to a significant increase in PtTMPyP4 fluorescence, which was used to further investigate binding between PtTMPyP4 and human telomeric DNA. A representative titration of PtTMPyP4 with Tel22 is shown in Fig. 4a. The data were analyzed using Direct Fit applying different binding models (5:1, 6:1, 7:1, etc.) with the best fit obtained for 7:1 PtTMPyP4 to Tel22 binding. The binding constant obtained, (1.8 ± 0.3) × 10^6^ M^−1^ and the stoichiometry are in good agreement with the data from UV–Vis titrations.

In order to determine the extent to which the porphyrin is selective for GQ over duplex DNA, PtTMPyP4 was also titrated with CT DNA under conditions identical to those used for Tel22. The results, shown in Fig. 4b, indicate that the fluorescence of PtTMPyP4 increases in the presence of CT DNA but to a substantially lower extent as compared to Tel22. Data fitting result in a 1:1 binding model and a binding constant of (1.1 ± 0.5) × 10^6^ M^−1^ per base-pair in CT DNA. It is important to point out that while the concentration of Tel22 is measured per DNA strand (22 nt), concentration of CT DNA is expressed per base-pair.

Binding constants and stoichiometry of binding determined for PtTMPyP4 and for PdTMPyP4 in 5K buffer are comparable to the binding constants reported for TMPyP4 or its other metallated analogues. Literature binding constants for TMPyP4 vary greatly within at least three orders of magnitude and the stoichiometry varies between 2 and 4 depending on the DNA sequence, buffer composition, and experimental method used [39, 49, 53]. For example, binding of TMPyP4 and its Ni(II) derivative to human telomeric DNA is characterized by *K*_a_ of ~10^6^ M^−1^; binding of Mn(III)TMPyP4 is an order of magnitude stronger with *K*_a_ of ~10^7^ M^−1^ (all measured via SPR). The latter porphyrin was also shown to be the most selective of the three [40]. Binding of TMPyP4 to Tel22 in 150 mM K^+^ buffer was described using three binding events with two strong binding constants, *K*_1_ = 7 × 10^8^, *K*_2_ = 6 × 10^7^, and *K*_3_ = 4 × 10^4^ M^−1^ [39]; the binding parameters were measured via ITC. In the same study binding of Cu(II)-, Ni(II)-, Co(III)- and ZnTMPyP4 was also characterized. The first two complexes display two binding events and strong binding with *K*_a_ (CuTMPyP4) = 2 × 10^10^ M^−1^, and *K*_a_ (NiTMPyP4) = 7 × 10^7^ M^−1^ for the strongest binding event. Binding of ZnTMPyP4 is weaker with *K*_a_ = 8 × 10^5^ M^−1^ and binding of Co(III)TMPyP4 to Tel22 is weak with *K*_a_  = 1 × 10^5^ M^−1^ most likely due to the presence of axial water ligands in these two complexes [39]. Cu(II)TMPyP4 binds to parallel tetrastranded quadruplexes [dT_4_G_4_T_4_]_4_ and [dT_4_G_8_T_4_]_4_ with *K*_a_ of (0.1–5) × 10^7^ M^−1^ depending on the model used [35]. Finally, ZnTMPyP4 binds to a bimolecular [(dTAGGG)_2_]_2_ quadruplex with binding stoichiometry of 2 and *K*_a_ of 6 × 10^6^ M^−1^ (from UV–Vis titrations) [36]; ZnTMPyP4 also was reported to bind to Pb^2+^-induced Tel22 quadruplex with binding stoichiometry of 2; *K*_a_ was not reported [54]. Overall, TMPyP4 and its metallated derivatives (including Pt- and PdTMPyP4) display strong binding to human telomeric DNA. While every study agrees on the end-stacking as the most efficient binding mode with high value of *K*_a_, the high stoichiometry is usually explained by additional binding modes. Lewis, et al. suggest intercalation [39]; while our values of hypochromicity and red shift as well as the presence of CD-induced signal (see below) could be interpreted as the result of intercalation, this binding mode is not favored in the quadruplex field as it has never been observed experimentally in any structural studies. Groove binding was suggested in another report [40] and was used also to explain the unusually high selectivity of Mn(III)TMPyP4.

### Stabilizing properties of PtTMPyP4 and PdTMPyP4 and selectivity toward Tel22 assessed through FRET

Fluorescent energy resonance transfer (FRET) assay is a benchmark technique in the quadruplex field to probe the stability and selectivity of quadruplex ligands [55]. To ascertain the stabilizing capabilities of Pt- and PdTMPyP4 toward the human telomeric quadruplex, we used its fluorescently labeled version, F21D. In K^+^ buffer, F21D forms a hybrid intramolecular GQ and in 50Na buffer F21D forms an antiparallel GQ, just like its unlabeled counterpart, Tel22. The secondary structure of F21D was confirmed by CD and thermal denaturation studies (TDS), and both signatures were similar to those for Tel22 [56]. In FRET, the stability of F21D (0.2 µM) is measured in the presence of increasing amounts of ligand and the data are usually reported in the form of ΔTm, the change in melting temperature for DNA–ligand complex as compared to Tm of DNA alone.

**Fig. 5:**
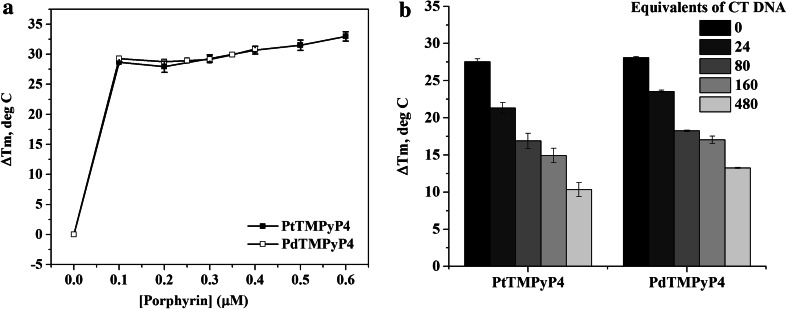
Stabilizing ability and selectivity of Pt- and PdTMPyP4 via FRET in 5K buffer. **a** Increase in the stabilization temperature of 0.2 µM F21D as a function of porphyrin concentration. **b** Selectivity of Pt- and PdTMPyP4 toward F21D in the presence of duplex competitor, CT DNA, amount of which is specified in the legend. Porphyrins and F21D were used at 0.2 μM in the selectivity experiments

In a conventional FRET setup, up to 20-fold ligand is added to DNA, but in our case, this amount of ligand led to melting curves with fluorescence values below 0.5 at the maximum temperature of 95 °C (Figure S5), suggesting incomplete quadruplex melting (ligand-stabilized GQ has a Tm > 95 °C). Therefore, the experiments were repeated with a reduced amount of porphyrins, only up to 3 equivalents, Fig. 5a. Observed stabilization temperatures, ΔTm, are 30.7 ± 0.6 °C for PtTMPyP4 and 30.9 ± 0.4 °C for PdTMPyP4 in 5K at 0.4 μM ligand (2:1 porphyrin-to-GQ). Both Pt- and PdTMPyP4 stabilize F21D more strongly than TMPyP4, CuTMPyP4, ZnTMPyP4 [36] and *N*-methyl mesoporphyrin IX (NMM) [56]. In order to ensure that the observed increase in fluorescence with increased temperature is due to unfolding of the fluorescently labeled quadruplex and not due to the natural fluorescence of the dissociated metalloporphyrin, FRET experiments were repeated using non-fluorescent DNA, Tel22, instead of F21D (Figure S6), and showed the baseline fluorescence only.

FRET experiments conducted in the presence of DNA competitor can provide information about ligands selectivity for GQ vs. other DNA structure. Because duplex DNA is present in excess in the cell, double-stranded calf thymus DNA was selected as the competitor. Selectivity for GQ is determined by finding the melting temperature of a GQ-forming sequence in the presence of increasing concentrations of unlabeled CT DNA at fixed concentration of ligand. Ligands selective for GQ DNA should bind to the GQ in the presence of the competitor as strongly as they bind to GQ alone. Thus, unchanging Tm observed with increasing CT DNA concentration is consistent with excellent selectivity. Our results, Fig. 5b, show that the stabilization temperature of F21D in the presence of 1 equivalent of porphyrins decreases upon the addition of up to 480 fold excess of CT DNA, indicating that both porphyrins are only modestly selective for quadruplex vs. duplex DNA. To quantify the selectivity, we used the selectivity ratio, defined as the excess of competitor necessary for a 50 % decrease in the observed stabilization temperature. Pt- and PdTMPyP4 display selectivity ratios of 150 and 330, respectively, in 5K. These value are similar to those obtained for TMPyP4 (selectivity ratio of 300) and ZnTMPyP4 (selectivity ratio of 100) [36], but much lower than that of NMM (high selectivity ratio, well above 480) [56]. Modest selectivity of tetracationic porphyrins (TMPyP4, Zn-, Pt- and PdTMPyP4) is most likely a result of the strong electrostatic interactions between the 4+ charged porphyrin and negatively charged DNA (whether it is quadruplex, duplex or other DNA topology, also see below). Overall, FRET studies suggest that Pt- and PdTMPyP4 are extremely robust and moderately selective stabilizers of mixed-hybrid F21D quadruplex in K^+^ condition.

### ‘Light-switch’ effect of PtTMPyP4 in the presence of a variety of DNA structures

**Fig. 6:**
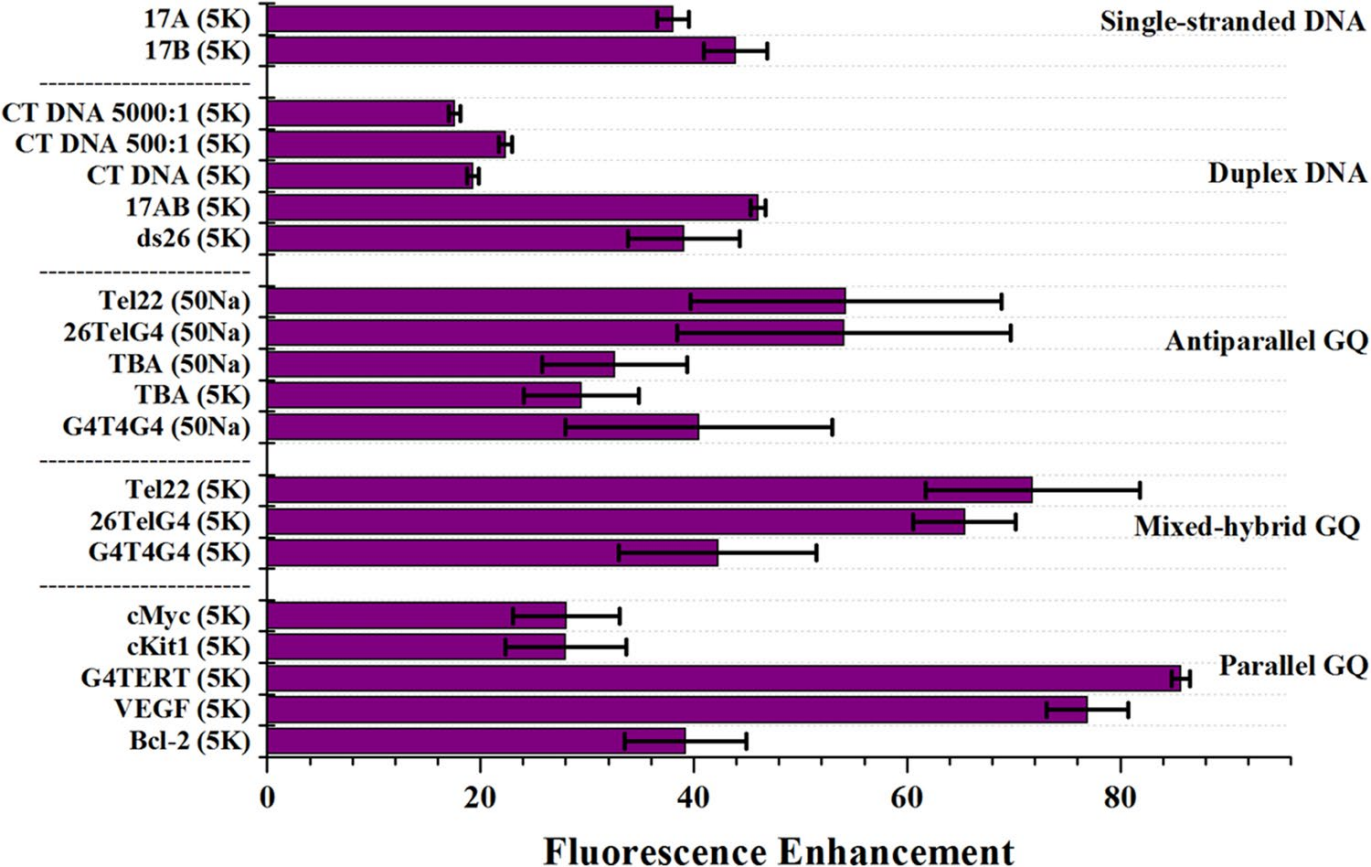
PtTMPyP4 fluoresces in the presence of a variety of DNA secondary structures. Fluorescence enhancement is determined relative to the fluorescence of PtTMPyP4 alone. The DNA-to-porphyrin ratio is 50:1 for all DNA except for CT, for which it is 500:1 and 5000:1. The buffer used is indicated in parentheses next to the name of the DNA sequence

Fluorescent titration studies, discussed earlier, made use of ‘light-switch’ property of PtTMPyP4 whose emission increases significantly upon binding to DNA [48]. Fluorescence enhancement is an ideal method to investigate the selectivity of PtTMPyP4 toward a variety of DNA secondary structures. Our laboratory has used this method to demonstrate an excellent selectivity of another porphyrin, NMM, toward quadruplexes [57]. Here we tested the effect of single-stranded DNA (17A, 17B), duplex DNA (CT DNA, ds26, 17AB) and quadruplex DNA (oncogene promoters: Bcl-2, cMyc, cKit1, VEGF, G4TERT; and telomeric DNA: Tel22, 26TelG4, G_4_T_4_G_4_) on fluorescence of PtTMPyP4. The list of all the DNA sequences can be found in Table 1. For quadruplexes, the experiments were conducted both in K^+^ buffer, in which quadruplexes are usually (but not always) parallel, and in Na^+^ buffer, in which an antiparallel geometry is expected. The results (Fig. 6; Table 1) demonstrate that PtTMPyP4 displays ‘light-switch’ effect in the presence of all DNA secondary structures. Overall and on average, the fluorescence enhancement is the highest in the presence of quadruplexes, notably VEGF, G4TERT and telomeric DNA (Tel22 and 26TelG4). PtTMPyP4 does not differentiate between different quadruplex folds, as we observed earlier with NMM [57]. In summary, PtTMPyP4 displays a moderate selectivity for quadruplex DNA vs. other DNA structures as was already shown in FRET assay (see above). This is the most comprehensive study of ‘light-switch’ effect displayed by PtTMPyP4 in the presence of a wide variety of DNA secondary structure. This study suggests broad selectivity for PtTMPyP4.

### PtTMPyP4–Tel22 binding interactions probed by circular dichroism

**Fig. 7:**
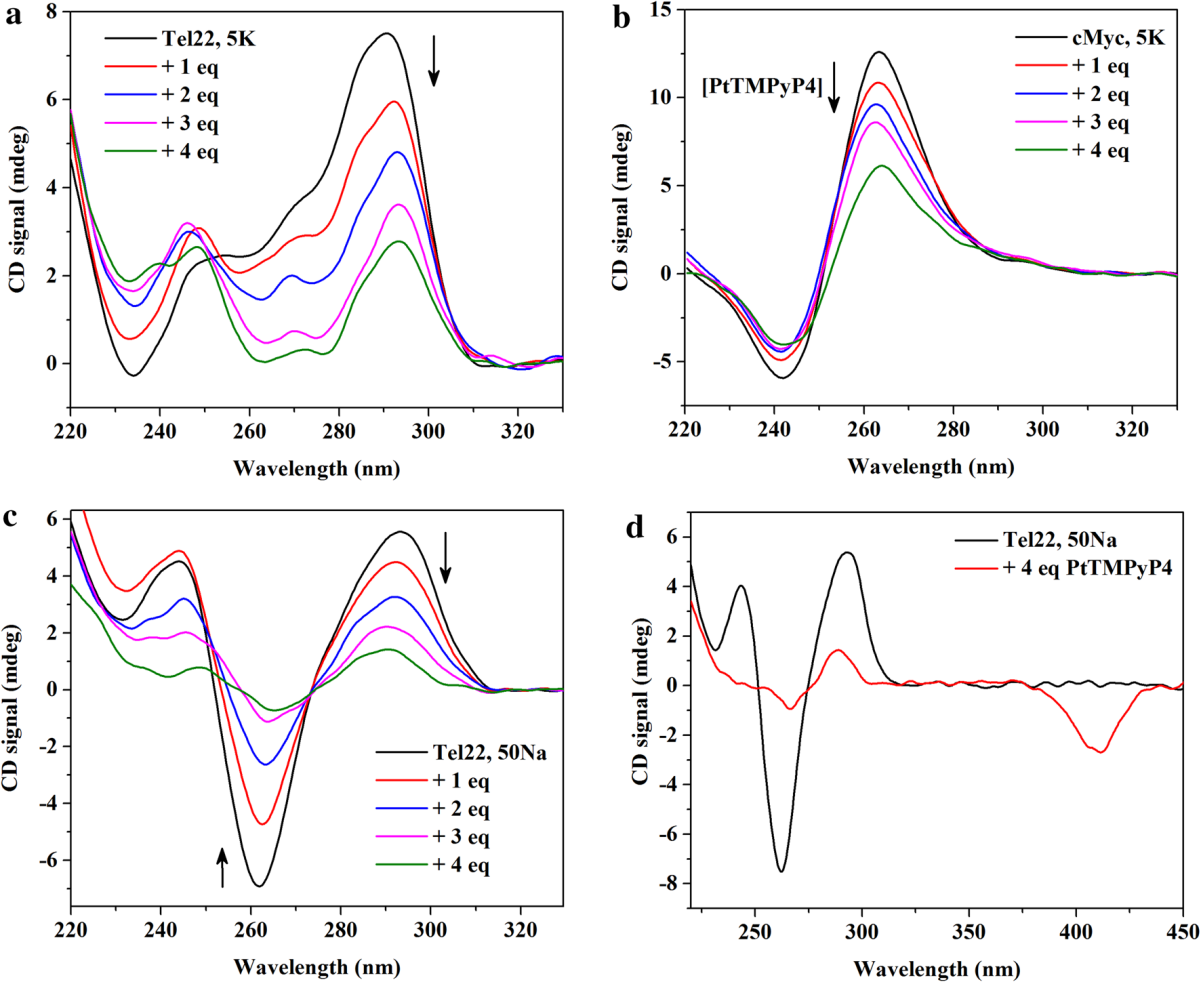
PtTMPyP4 reduces CD signal of DNA quadruplexes. **a** Representative titration of 2 µM Tel22 in 5K buffer; **b** representative titration of 2 µM cMyc in 5K buffer, and **c** representative titration of 2 µM Tel22 in 50Na buffer. **d** Induced CD signal in the porphyrin Soret band at 410 nm in the CD spectrum of Tel22 in 50Na buffer upon addition of 4 equivalents of PtTMPyP4 (same as the *green line* in part **c**)

To determine if PtTMPyP4 affects the topology of a quadruplex, CD titration and annealing experiments were performed using three representative quadruplexes: cMyc in 5K (parallel topology), Tel22 in 5K (mixed-hybrid topology) and Tel22 in 50Na (antiparallel topology). The cMyc oncogene promoter is a transcriptional regulator, overexpressed in up to 80 % of solid tumors [3]. This DNA displays the typical parallel quadruplex CD signature with a peak at 265 nm and a trough at 244 nm. In 5K buffer, human telomeric DNA, Tel22, displays peaks at 294 and 255 nm and a trough at 235 nm, a signal consistent with the mixed-hybrid structure [17, 58]. The CD of Tel22 in 50Na buffer consists of a peak at 294 nm and a trough at 260 nm, which is a typical signature of an antiparallel GQ. In all cases and indiscriminately, addition of PtTMPyP4 preserves the overall CD signature but leads to a decrease in CD signal intensity (Fig. 7). Signal decrease is concentration dependent. Similar decrease in CD signal intensity was observed upon titration of Tel22 in K^+^ and in Na^+^ buffers with TMPyP4 and its Cu(II)- and Ni(II)-derivatives. On contrary, when Co(III)TMPyP4 or Zn(II)TMPyP4 were added to Tel22, its CD signal did not change [39]. The decrease in CD signal intensity could be due to the preferential interaction of these porphyrins with single-stranded DNA as well as to the partial precipitation of DNA at high porphyrin/DNA ratios. It is conceivable that PtTMPyP4 could replace (unfold) one of the tetrads, leading to decrease in the tetrad stacking (hence decrease in CD signal) at the same time stabilizing the quadruples (hence higher Tm).

CD titrations are run in the kinetic regime (with fast additions of the porphyrin), thus, it is possible that insufficient time is allowed for a ligand to affect the observed CD signature. To test the effect of PtTMPyP4 on the three DNA topologies under the equilibrium conditions, CD annealing experiments were employed: DNA samples were annealed in the presence of PtTMPyP4 and equilibrated overnight after which the CD scans were collected. The results, shown in Figure S7, are consistent with the titration studies, where shape of the signals is preserved but the intensity is attenuated. We also checked for the presence of induced CD signal, which indicates a strong interaction between DNA and the porphyrin. Indeed, negative induced signal was observed in the CD spectra of antiparallel Tel22 in the visible region of CD spectra at ~410 nm (close to PtTMPyP4 Soret band) upon addition of 4 equivalents of PtTMPyP4 (Fig. 7d). Negative induced CD signal was also observed upon CD titration of B-form pBluescript II plasmid DNA with Pt- and PdTMPyP4 [48]. In this latter case, intercalation of the porphyrin into the DNA duplex was used as an explanation for the observed induced CD signal. Titration of cMyc or Tel22 in 5K with PtTMPyP4 led to no (or very weak) induced CD signal. Absence of induced signal in the porphyrin’s Soret region of CD spectra does not necessarily signify an absence of the interaction between the porphyrin and DNA, as NMM was shown to interact with a variety of quadruplex structures but displayed no (or weak) induced CD signal [59]. Overall, the presence of induced CD signal signifies close interaction (involving most probably efficient overlap of π–π systems and consistent with end-stacking) between the ligand and GQ DNA; absence of induced CD signal does not exclude the interaction, but might indicate a different binding mode (via loops or grooves).

## Concluding remarks

In this work, we investigated the interaction between two metallated derivatives of TMPyP4, Pt- and PdTMPyP4, and a variety of GQ DNA structures with the main focus on human telomeric DNA, Tel22. Via a combination of CD, UV–Vis, and FRET studies we were able to demonstrate that Pt- and PdTMPyP4 interact effectively with GQ DNA and stabilize the quadruplex topology. The binding mode most probably involves end-stacking on both sides of Tel22 with possible additional binding of PtTMPyP4 to already bound porphyrin molecules (i.e., aggregation). The aggregates dissociate in the presence of additional Tel22. Pt- and PdTMPyP4 behave rather similarly toward quadruplex DNA, which is not surprising given that both ligands adopt similar geometry and both metal centers contain the same number of valence electrons (d^8^) and have similar ionic radii, 74 pm and 78 pm for Pt(II) and Pd(II), respectively. PtTMPyP4 has broad selectivity toward DNA and its fluorescence can be ‘turned on’ by many DNA structures including single-stranded DNA, DNA duplex and a variety of DNA quadruplexes. Quadruplexes, however, lead to the highest fluorescence enhancement. Modest selectivity for quadruplex vs. other DNA structures is an important flaw in the properties of these molecules, but their exceptional stabilizing ability (by >30 °C at 2 equivalents; better that stabilizing ability of TMPyP4) combined with high fluorescence enhancement in the presence of quadruplex DNA make these molecules valuable quadruplex ligands.

## Electronic supplementary material

Supplementary material 1 (PDF 843 kb)

## Abbreviations

CT: Calf thymus DNA
GQ: Guanine quadruplex DNA
PtTMPyP4: Pt(II) 5,10,15,20-tetrakis (*N*-methyl-4-pyridyl) porphyrin
PdTMPyP4: Pd(II) 5,10,15,20-tetrakis (*N*-methyl-4-pyridyl) porphyrin
Tel22: Human telomeric DNA repeat model sequence
