# Supplementary material for: Investigation of the interactions between Pt(II) and Pd(II) derivatives of 5,10,15,20-tetrakis (N-methyl-4-pyridyl) porphyrin and G-quadruplex DNA
Source: J Biol Inorg Chem. 2016 Jan 9;21:227–39. doi: 10.1007/s00775-015-1325-8 (PMC4801998; doi:10.1007/s00775-015-1325-8)
Supplement: Supplementary file 1 — Supplementary material 1 (PDF 843 kb) [file 775_2015_1325_MOESM1_ESM.pdf]

*Electronic Supplementary Material*

**Investigation of the interactions between Pt(II) and Pd(II) derivatives of 5,10,15,20-tetrakis (*N*-methyl-4-pyridyl) porphyrin and G-Quadruplex DNA**

Navin C. Sabharwal[1#], Oscar Mendoza[2,3], John M. Nicoludis[1&], Thomas Ruan[1], Jean-Louis Mergny[2,3], and Liliya A. Yatsunyk[1]

[1]*Department of Chemistry and Biochemistry, Swarthmore College, 500 College Ave., Swarthmore, PA 19081 USA;* [2]*INSERM U869, IECB, F-33600 Pessac, France.* [3]*Université de Bordeaux, Bordeaux, France.*

Present address: [&]Department of Chemistry and Chemical Biology, Harvard University, 12 Oxford St., Cambridge, MA, 02138 USA; # Lerner College of Medicine, Cleveland Clinic, 9500 Euclid Ave., Cleveland, OH, 44195 USA

✉ Corresponding author: Liliya A. Yatsunyk; E-mail: lyatsun1@swarthmore.edu; Fax: 610 328 7355

**PtTMPyP4 and PdTMPyP4 do not aggregate in aqueous solutions.** Porphyrin aggregation due to π-π stacking is a concern when studying their interaction with DNA as it greatly complicates the system under investigation. Simple UV-vis dilution experiments can test Beer's Law and identify possible aggregation within experimental range of concentrations. Linear increase in the absorbance values with concentration usually suggests absence of aggregation, although it could also signify the existence of stable aggregates (dimers, trimers, etc) that do not dissociate under experimental conditions. For PtTMPyP4 absorbance at Soret maximum increases linearly in the concentrations range from 0.5 to 20 µM in water; similar linear increase in the absorbance was observed for PdTMPyP4 in the concentration range of 0.5 and 50 µM (**Figure S1**). Note, most of the experiments in this work were done in the concentration range between 1 and 5 µM. Thus, under our experimental conditions, both porphyrins exist in a monomeric form, most likely due to their 4+ charge.

## Figure S1

**Figure S1. PtTMPyP4 and PdTMPyP4 do not aggregate in aqueous solutions.** Results of the aggregation study for PtTMPyP4 in water and PdTMPyP4 in 5K buffer. The extinction coefficient for PtTMPyP4 was determined to be $1.73 \times 10^5\ M^{-1}cm^{-1}$ at 401 nm in water, as compared to $\varepsilon_{401} = 1.72 \times 10^5\ M^{-1}cm^{-1}$ (*1*) and the extinction coefficient of PdTMPyP4 was determined to be $1.67 \times 10^5\ M^{-1}cm^{-1}$ at 418 nm in 5K, as compared to $\varepsilon_{418} = 1.68 \times 10^5\ M^{-1}cm^{-1}$ (*2*).

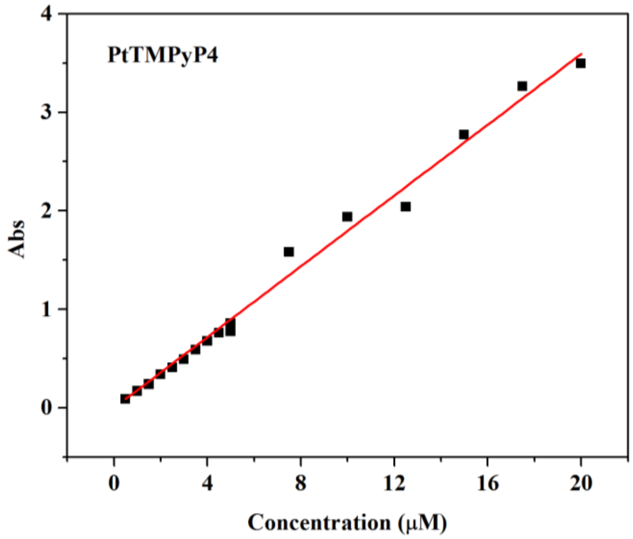


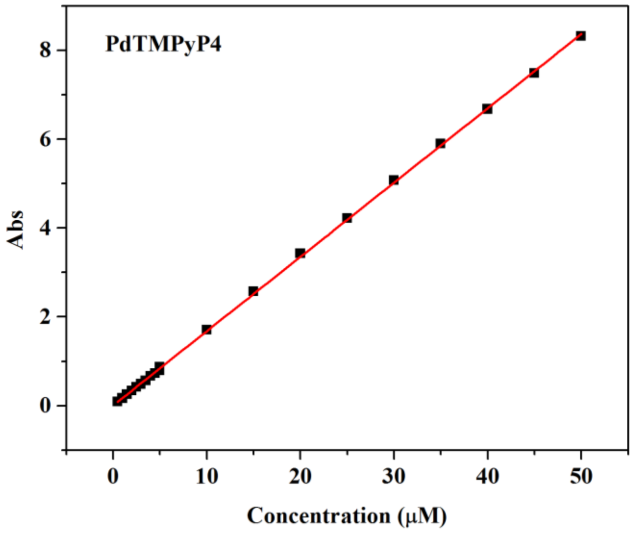

# Figure S2

**Figure S2. Titration of PdTMPyP4 with Tel22 in 5K.** (**a**) Representative UV-vis data for titration of 2.87 μM PdTMPyP4 with 30 μM Tel22 in 5K buffer. (**b**) Scatchard plot of *r*/[PdTMPyP4]free vs. *r*, where r is a binding ratio that equals to $[PdTMPyP4]_{bound}$/[DNA]. Scatchard plot yielded stoichiometry of 6.5:1 porphyrin to DNA and a binding constant of $(0.78 \pm 0.03)\times10^{7}\ M^{-1}$. (**c**) Direct fit of titration data at specified wavelengths. Dashed lines correspond to the 95% confidence interval. Direct fit yielded a binding constant of $(1.1 \pm 0.3)\times10^{7}\ M^{-1}$ when 7:1 porphryin:DNA binding model was used. Data from Scatchard and direct fits are in excellent agreement.

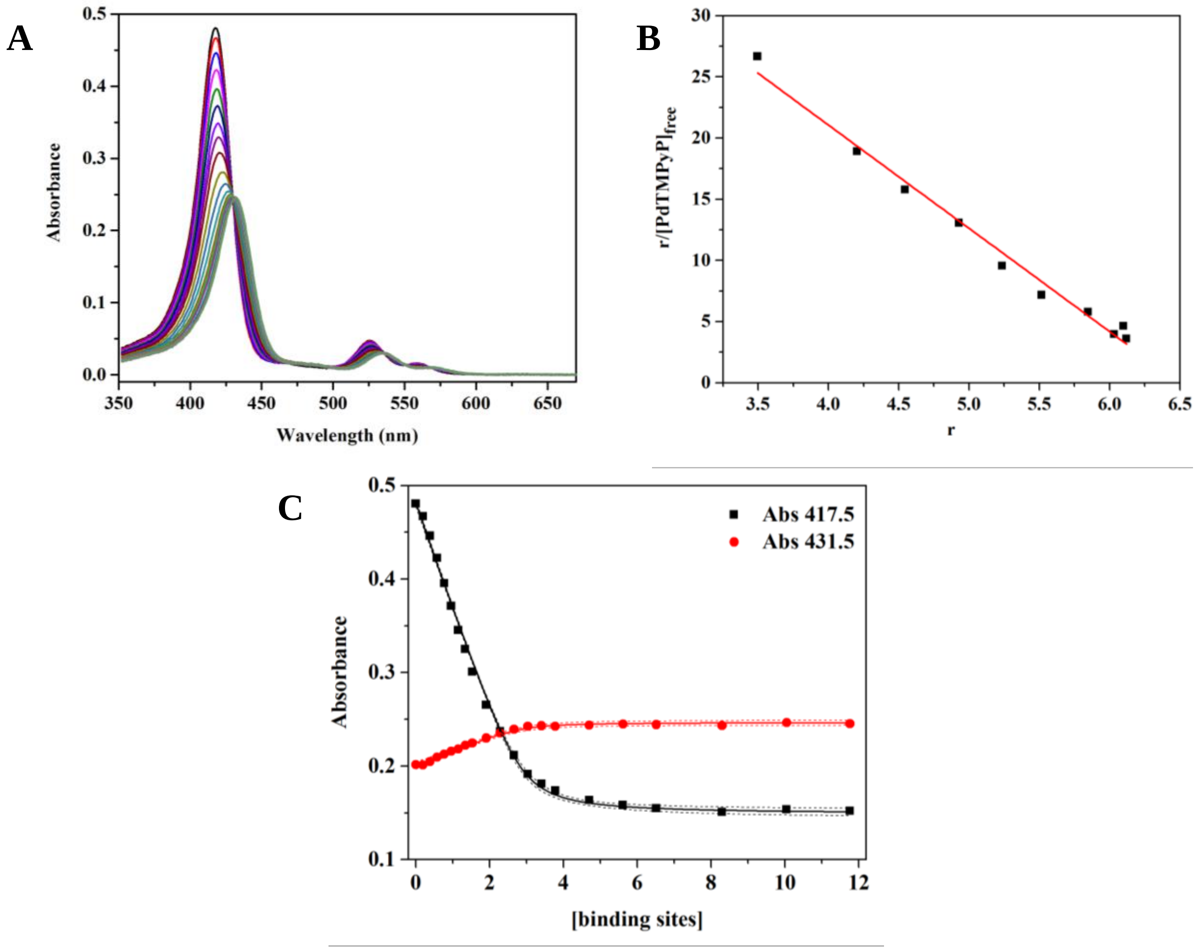

## Figure S3

**Figure S3. Titration of PtTMPyP4 with Tel22 in KPi buffer (**10 mM KPi, pH 7.0, 50 mM KCl). (**a**) Representative UV-vis data for titration of 1.2 μM PtTMPyP4 with 33.0 μM Tel22. Analysis of three independent titration data led to a red-shift of 13.8 ± 0.3 nm and a hypochromicity of 45 ± 2% (**b**) Scatchard plot of *r*/[PtTMPyP4]free vs. *r*, where r is a binding ratio that equals to $[PtTMPyP4]_{bound}/[DNA]$. Scatchard plot yielded stoichiometry of (6.7 ± 0.7):1 porphyrin to DNA and a binding constant for the titration depicted below of $(1.4 \pm 0.7)\times10^{8}$ $M^{-1}$. The binding constant based on three independent experiments is $(2.8 \pm 2.6)\times10^{8}$ $M^{-1}$. (**c**) Direct fit of the titration data at specified wavelengths. Direct fit yielded a binding constant of $(1.4 \pm 0.2)\times10^{8}$ $M^{-1}$ when a binding stoichiometry of 6.6 porphyrin to DNA was used. Note, interestingly, the biding affinity in KPi buffer is significantly higher as compared to the same data in 5K buffer and could possibly be explained by the difference in ionic strength of ~60 mM for KPi and 110 mM for 5K buffers. Usually, increase in the ionic strength leads to weakening of interactions that have significant electrostatic component.

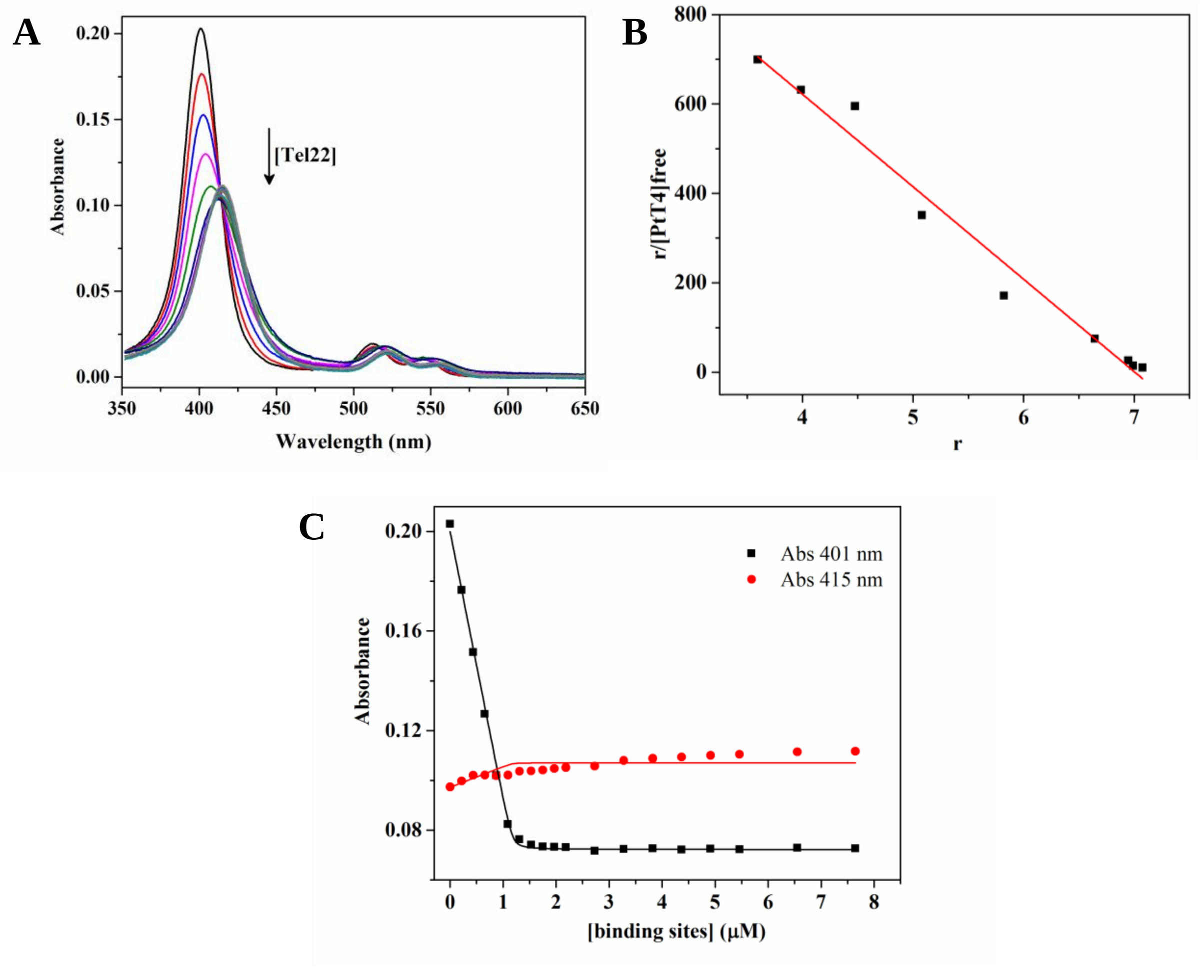

**Continuous variation analysis for PtTMPyP4 in KPi buffer.** Job plot experiments were conducted as described in the main text using ~3-5 µM PtTMPyP4 and Tel22 in KPi buffer. Additionally, Job plot was also conducted in the narrow range of mole fraction, in order to visualize multiple binding events. Three independent experiments yielded binding stoichiometry of 6.6 ± 0.2 in good agreement with the Scatchard analysis data and direct fit, **Figure S3**. However, the experiment was repeated with the increased resolution revealing possibly two binding events with the stoichiometries of 3.6 ± 0.1 and 8.4 ± 0.4. This data could be interpreted as 3 to 4 molecules of PtT4 bind Tel22 with high affinity, following additional binding of another 4 molecules of the porphyrin. This second step could signify i) binding to additional binding sites on Tel22, which is rather difficult to imagine; ii) non-specific binding between negatively charged DNA and positively charged porphyrin molecule, yet, reproducible binding stoichiometry argues against this case; and iii) stacking of additional porphyrins onto already bound porphyrin molecules. To test this latter possibility, we examined the potential charge transfer of two metal complexes by looking for the metal-metal-to-ligand-charge transfer (MMLCT) band in the near-infrared region, but did not observe any Pt-Pt interactions.

**Figure S4**

**Figure S4. Job's plots for PtTMPyP4 with Tel22 in KPi**. **(a)** Overlay of the data from three separate titrations yielding averaged binding stoichiometry of 6.6 ± 0.2. Absorbance difference at 399 nm was plotted as a function of molar fraction of the porphyrin. **(b)** Higher resolution Job's plot data suggesting two binding ratios of 3.6 ± 0.1 and 8.4 ± 0.4. Data are from the same titration at three different wavelengths indicated in the legend.

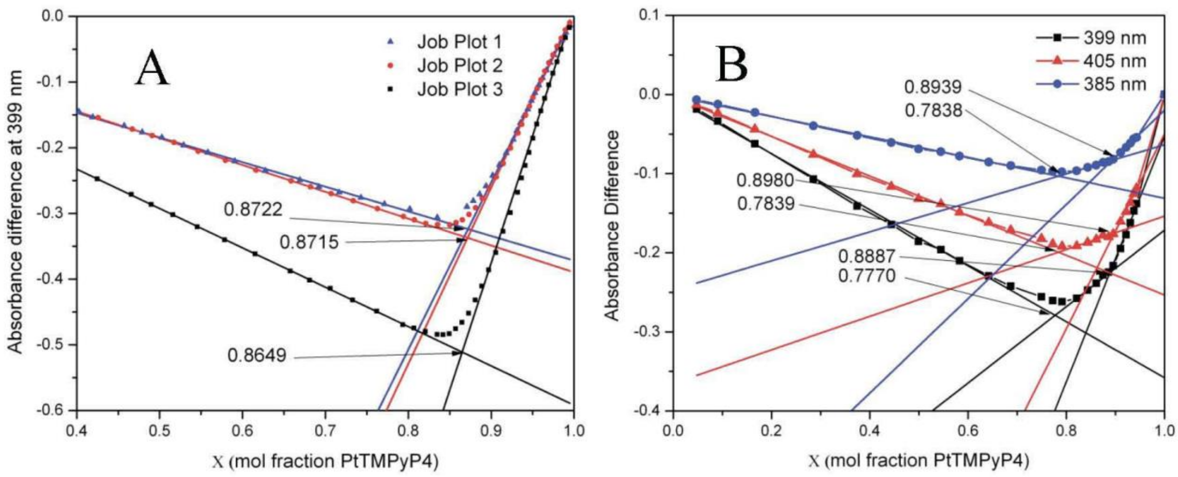

# Figure S5

**Figure S5. FRET melting of F21D in the presence of up to 4 μM of (a) PtTMPyP4 and (b) PdTMPyP4**. The low fluorescence values of F21D in the presence of > 0.4 μM ligand might indicate incomplete melting of the quadruplex. Experiments were completed in 5K buffer.

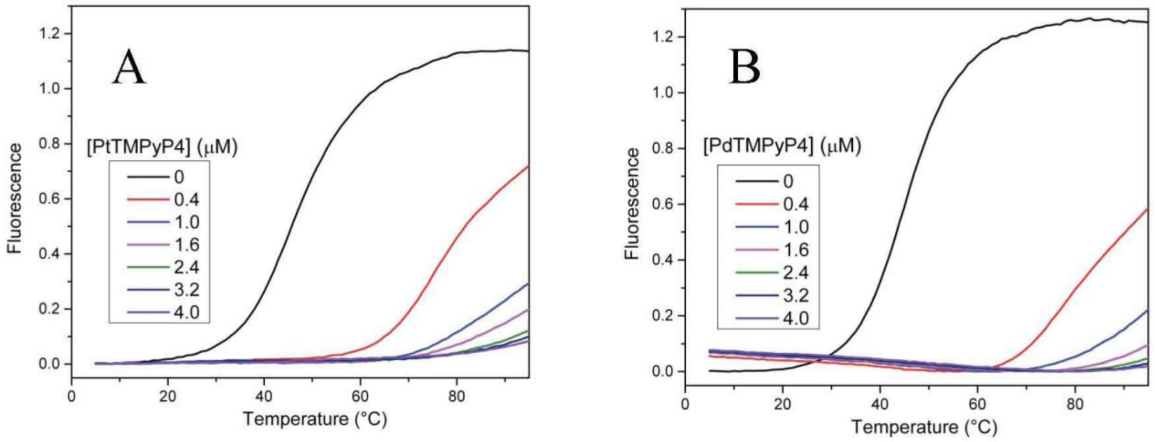

**Figure S6**

**Figure S6. Control experiments for FRET melting.** Absence of fluorescence emitted by (**a**) PtTMPyP4 and (**b**) PdTMPyP4 in the presence of unlabeled human telomeric DNA, Tel22, indicates that the natural porphyrin fluorescence is not responsible for the fluorescence changes observed in FRET experiments.

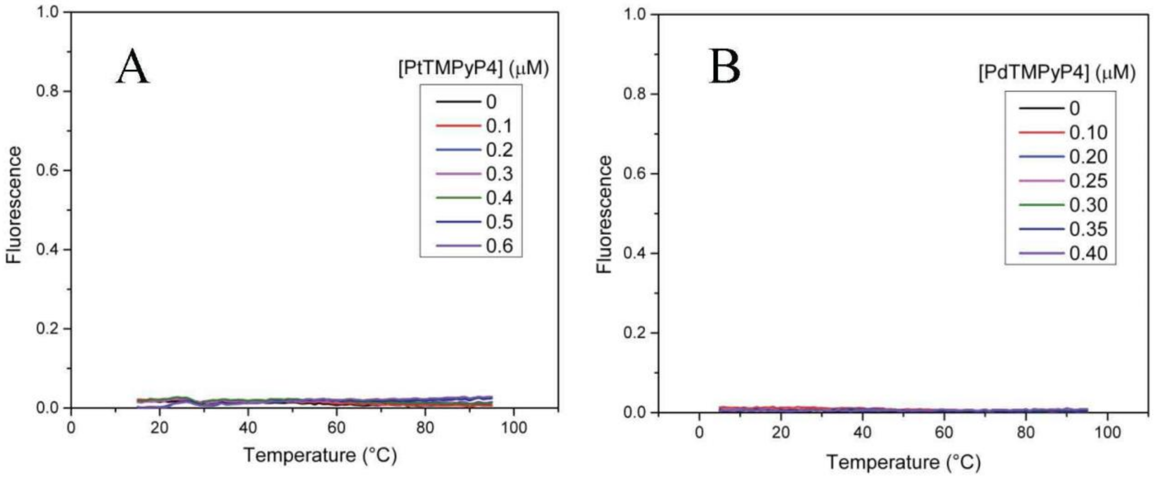

## Figure S7

**Figure S7. CD annealing studies** for PtTMPyP4 and representative quadruplex strucures (a) cMyc in 5K buffer (parallel structure), (b) G4TERT in 5K buffer (mixed-hybrid structure), and (c) Tel22 in 50Na buffer (anti-parallel structure). Porphyrin was annealed with DNA in a 2:1 ratio at 90 °C for ten minutes, cooled slowly to room temperature over 3 hours, and then incubated at 4 °C overnight to ensure equilibration before CD scans were taken.

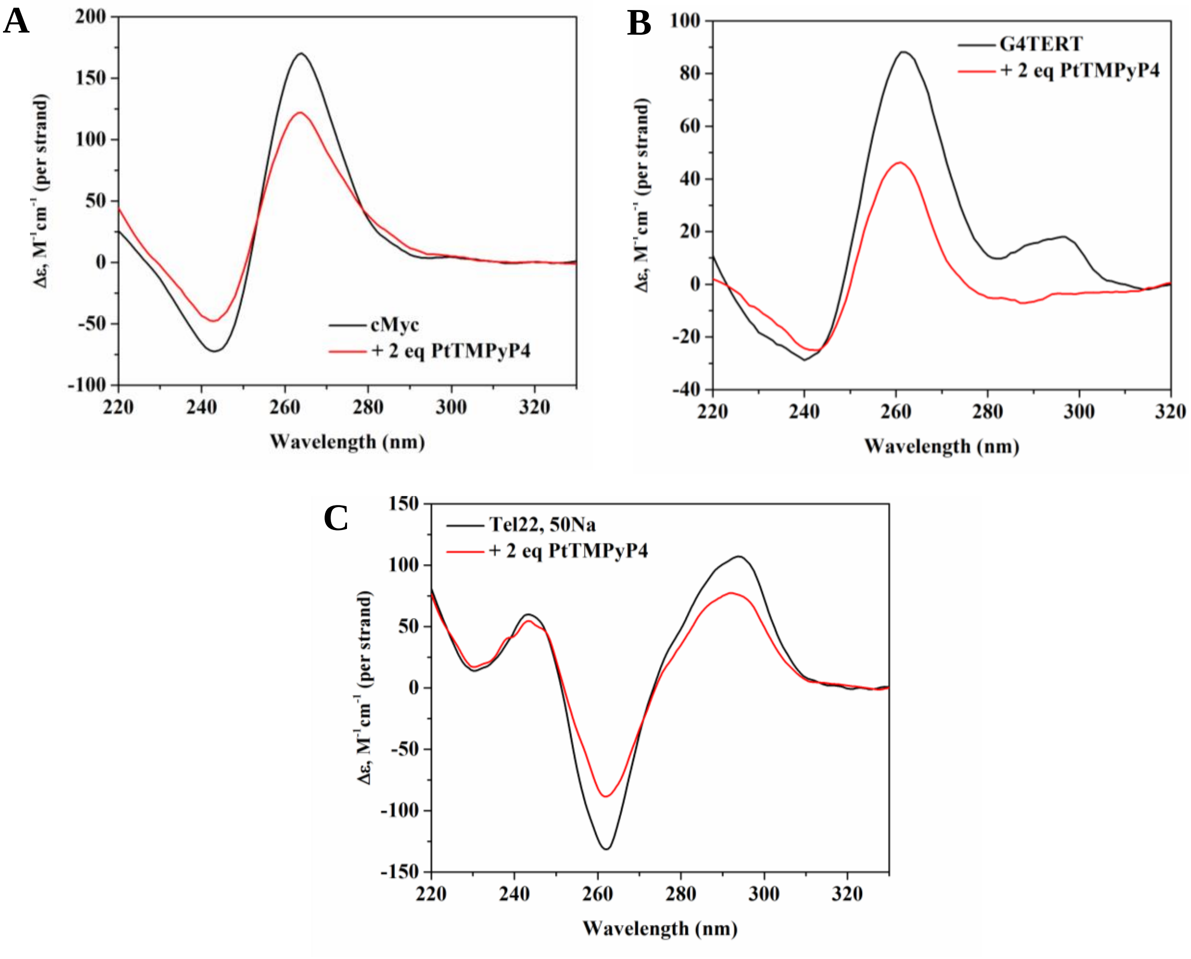

## References


1. Pasternack, R. F., Brigandi, R. A., Abrams, M. J., Williams, A. P., and Gibbs, E. J. (1990) Interactions of porphyrins and metalloporphyrins with single-stranded poly(dA), *Inorg. Chem. 29*, 4483-4486.
2. Roza-Fernández, M., Valencia-González, M. J., and Díaz-García, M. E. (1997) Room-temperature phosphorescent palladium-porphine probe for DNA determination, *Anal Chem 69*, 2406-2410.
